# Reduced cilia frequencies in human renal cell carcinomas versus neighboring parenchymal tissue

**DOI:** 10.1186/2046-2530-2-2

**Published:** 2013-01-31

**Authors:** Sander G Basten, Sven Willekers, Joost SP Vermaat, Gisela GG Slaats, Emile E Voest, Paul J van Diest, Rachel H Giles

**Affiliations:** 1 Department of Nephrology and Hypertension, University Medical Center Utrecht, Heidelberglaan 100, 3584, CX, Utrecht, The Netherlands; 2 Department of Medical Oncology, University Medical Center Utrecht, Universiteitsweg 100, 3584, CG, Utrecht, The Netherlands; 3 Department of Pathology, University Medical Center Utrecht, Heidelberglaan 100, 3584, CX, Utrecht, The Netherlands

**Keywords:** Cilia, Kidney, Clear cell renal cell carcinoma, Chromophobe RCC, Papillary RCC, Oncocytoma, Histology

## Abstract

**Background:**

Cilia are essential organelles in multiple organ systems, including the kidney where they serve as important regulators of renal homeostasis. Renal nephron cilia emanate from the apical membrane of epithelia, extending into the lumen where they function in flow-sensing and ligand-dependent signaling cascades. Ciliary dysfunction underlies renal cyst formation that is in part caused by deregulation of planar cell polarity and canonical Wnt signaling. Renal cancer pathologies occur sporadically or in heritable syndromes caused by germline mutations in tumor suppressor genes including *VHL*. Importantly, Von Hippel-Lindau (VHL) patients frequently develop complex renal cysts that can be considered a premalignant stage. One of the well-characterized molecular functions of VHL is its requirement for the maintenance of cilia. In this study, tissue from 110 renal cancer patients who underwent nephrectomy was analyzed to determine if lower ciliary frequency is a common hallmark of renal tumorigenesis by comparing cilia frequencies in both tumor and adjacent parenchymal tissue biopsies from the same kidney.

**Methods:**

We stained sections of human renal material using markers for cilia. Preliminary staining was performed using an immunofluorescent approach and a combination of acetylated-α-tubulin and pericentrin antibodies and DAPI. After validation of an alternative, higher throughput approach using acetylated-α-tubulin immunohistochemistry, we continued to manually quantify cilia in all tissues. Nuclei were separately counted in an automated fashion in order to determine ciliary frequencies. Similar staining and scoring for Ki67 positive cells was performed to exclude that proliferation obscures cilia formation potential.

**Results:**

Samples from renal cell carcinoma patients deposited in our hospital tissue bank were previously used to compose a tissue microarray containing three cores of both tumor and parenchymal tissue per patient. Cilia frequencies in a total of eighty-nine clear cell, eight papillary, five chromophobe renal cell carcinomas, two sarcomatoid renal tumors and six oncocytomas were determined. A marked decrease of primary cilia across renal cell carcinoma subtypes was observed compared to adjacent nontumorigenic tissue.

**Conclusions:**

Our study shows that cilia are predominantly lost in renal cell carcinomas compared to tissue of the tumor parenchyma. These results suggest that ciliary loss is common in renal tumorigenesis, possibly participating in the sequence of cellular events leading to malignant tumor development. Future therapies aimed at restoring or circumventing cilia signaling might therefore aid in current treatment efficacy.

## Background

Primary cilia are small hair-like organelles that generally extend from the apical plasma membrane and are almost ubiquitously expressed throughout the human body [1]. Cilia function as sensory organelles in response to extracellular stimuli, such as fluid flow (mechanosensation) in addition to mitogenic, morphogenic and olfactory factors. Ciliary signaling is pivotal during development and organ homeostasis [2]. Dysfunctional cilia underlie the development of a broad range of diseases, collectively known as ciliopathies. Hallmark disease syndromes that find their origin in cilia dysfunction include polycystic kidney disease (PKD), Bardet-Biedl syndrome (BBS), Meckel-Gruber syndrome (MKS) and nephronophthisis (NPHP) [3]. These syndromes have a partially overlapping disease spectrum and interestingly, one of the most frequently affected tissues is the kidney, which is characterized by a high prevalence of renal cyst formation [3]. The tubules that make up the nephron are highly ciliated and essential for regulating cell proliferation in response to fluid flow, as well as maintaining planar organization of the tissue [4]. Loss of cilia in the renal tubules has been described to induce a switch from non-canonical to canonical Wnt signaling that leads to inappropriate β-catenin activation, cell proliferation and loss of planar cell polarity, however there is some controversy concerning the exact mechanisms involved [5]. In general, loss of ciliary function marks the initiation of cyst formation eventually severely impairing renal function.

Kidney cancers can be subcategorized into several histopathological subtypes, of which renal cell carcinoma (RCC) is the most predominant [6]. Distinct RCC pathologies are clear cell (ccRCC) with an incidence of 75%, papillary (pRCC, 10 to 15%) and chromophobe (chrRCC, 5%) [7]. Closely resembling the histology of chromophobe RCC is the subtype renal oncocytoma with a prevalence of 5% [8,9]. The overall majority of kidney cancers is sporadic and 4% is attributable to heritable cancer syndromes [6]. The best-studied familial kidney cancer syndrome is Von Hippel-Lindau disease (VHL, MIM608537), predisposing to ccRCC development as well as extrarenal tumor development [10]. Interestingly, the *VHL* gene is also inactivated in up to 87% of sporadic clear cell RCCs [11]. Tuberous sclerosis (TSC) is associated with germline mutations in the *TSC1*/*Harmatin* (MIM605284) and *TSC2*/*Tuburin* (MIM191092) genes [12]. The renal pathology of TSC patients is predominantly benign renal angiomyolipoma (50 to 80%), although a minority develops ccRCC with an earlier onset of disease compared to the general population (3%) [13]. Birt-Hogg-Dubé syndrome (BHD) is a monogenic disorder caused by mutations in *FLCN*/*Folliculin*[14] (MIM607273). Renal tumors isolated from BHD patients are histologically diverse, predominantly consisting of chrRCC (34%) and hybrid oncocytoma/chromophobe (50%) neoplasms, and less frequently ccRCC (9%) [15]. Interestingly, the gene products of these heritable kidney cancer loci have all been directly linked with cilia function. As renal cysts are common features in VHL, TSC and BHD syndromes, a common hypothesis is that renal transformation originates from cystic lesions comprising a pretumorigenic stage in the natural disease course. In VHL patients, direct evidence of this transformation has been documented [16]. This is further supported by the observation that patients with acquired cystic kidney disease (ACKD) have an increased risk for RCC with incidences ranging from 2 to 5% [13], and the development of both cysts and RCC in mouse models for VHL disease [17], TSC [18] and BHD syndrome [19].

During the cell cycle, both the cilium and the mitotic spindle lay claim to the centrosome, rendering these processes mutually exclusive and cilia must therefore first be disassembled prior to cell division [20,21]. Collectively, these data raise the question of whether renal cell carcinomas sustain normal ciliary frequencies. One study in a small cohort of ccRCC indeed establishes some initial support for this hypothesis [22]. Here we analyze cilia frequency in renal tumor resections by immunohistoche-mistry for cilia axonemal marker acetylated-α-tubulin. In order to efficiently and reproducibly analyze a large number of patients in standardized conditions, we used a tissue microarray (TMA) of RCC biopsies, including eighty-nine ccRCC, eight pRCC, five chrRCC, two sarcomatoid renal tumors and six oncocytomas [23]. The TMA additionally includes tissue originating from the benign renal parenchyma surrounding the tumor, which we used to correlate the ciliary frequencies in RCC.

## Methods

### Patient material and tissue microarray

The tissues analyzed in this study were obtained from nephrectomy samples collected from patients at the University Medical Center Utrecht (UMCU) and part of the UMCU biobank. All patient samples conform to the medical and ethical guidelines applied by the UMCU and Dutch law. Selected tissues comprise a variety of RCC histologies; eighty-nine ccRCC, eight pRCC, five chrRCC, six oncocytomas and two sarcomatoid renal tumors (Table S1 in Additional file 1). The TMAs were previously constructed using an arrayer (Beecher Instruments, Sun Prairie, WI, USA) and three cores of 1 mm in diameter from the cancer tissue, and three cores of 1 mm in diameter from the healthy renal parenchyma tissue taken anywhere between 1 cm and 8 cm from the edge of the tumor were included. Every composite paraffin block included the same tumor and parenchyma tissue for normalization. All tissues and corresponding patient information used were well documented and previously categorized for histological subtype [23].

### Immunohistochemistry

TMA sections of 4 μm were processed as previously described [23]. Following deparaffination and rehydration, antigen retrieval was achieved by boiling in citrate buffer for 20 minutes and the sections were allowed to cool to room temperature over one hour on the bench. Endogenous peroxidase activity was blocked with 3% H_2_O_2_ for 15 minutes before blocking with 5% horse serum for one hour at room temperature. Acetylated-α-tubulin antibody from Sigma-Aldrich (St. Louis, MO, USA) (clone 6-11B-1 purified from mouse, 1:12,000) and Ki67 antibody from Dako (Glostrup, Denmark) (1:200) were used for primary staining overnight at 4°C. Secondary antibody staining was performed using anti-mouse HRP (1:100) and PowerVision (Immunologic, Duiven, The Netherlands) activated with H_2_O_2_ and stained with Novared. Serial sections were counterstained 15 to 30 seconds with hematoxylin and developed for 10 minutes in running tap water. To determine the amount of actively proliferating cells, the percentage of Ki67 positive cells were blindly scored by a pathologist together with an independent researcher (Table S1 in Additional file 1). The mean score of the three cores was used.

### Immunofluorescence

After deparaffination and rehydration, 4 μm sections were digested in protease XXIV (Sigma-Aldrich, 0.02 mg/ml in PBS, pH 7.3, 60 minutes at room temperature) [24]. After washing and blocking in 1% BSA in PBS, primary antibodies (mouse monoclonal acetylated-α-tubulin, clone 6-11B-1 (Sigma-Aldrich) 1:12,000, and rabbit polyclonal anti-pericentrin (Novus Biologicals, Littleton, CO, USA) 1:100) were incubated for 60 minutes at room temperature. After repeated washing in PBS, secondary antibodies are incubated for an additional 60 minutes at room temperature: goat anti-mouse conjugated to Cy5 (Millipore, Bedford, MA, USA) (1:100) and goat anti-rabbit-Cy3 (Life Technologies, Carlsbad, CA, USA) (1:100). Sections were washed again repeatedly, incubated in DAPI (diluted 1:5,000 in PBS) for 15 minutes and after a final round of washes in PBS, mounted with Fluoromount G. Stained sections are stored in the dark at 4°C until confocal imaging with a Zeiss LSM700 63x objective (Carl Zeiss Microscopy, Jena, Germany). Z-stacks covering 4 μm were taken until approximately 300 nuclei were scored blindly for cilia.

### Cilia counting and automated nuclei counting

Acetylated-α-tubulin-stained TMA sections were scanned at high resolution and analyzed using Aperio Imagescope (Aperio, Vista, CA, USA) [25]. Manual cilia counts were made from scans using a 40X digital zoom. The number of cilia counted was randomly double-checked by an additional researcher. Hematoxylin and eosin (H&E)-stained TMA sections were similarly scanned and individual tissue spots exported using Aperio Imagescope. Using Photoshop, a color selection range was matched to hematoxylin (as indicated in Figure S1 in Additional file 2). This selection was extracted from the original image to remove background and converted and inverted to an 8-bit file using ImageJ and built-in macros. Next, a binary image threshold was set between 10 and 255. The particle analysis was set for between a 25 and 300 pixel range and singularity 0 to 1.00 to identify single nuclei. To circumvent the problem of aggregated nuclei, a second and third particle analysis was performed using 300 to 550 and 550 to 5,000 pixel ranges to identify ‘two nuclei’ and ‘>’ respectively. The events identified by the various pixel analyses were summarized to calculate the total number of cells, where the event number identified ‘two nuclei’/300 to 550 pixel range was multiplied by two, and the ‘>’/550 to 5,000 pixel range was multiplied by five. The latter was chosen as, although visual observation shows a large variation, the number of multicellular clusters was low and five cells was the average number of cells present in these clusters. The number of events identified in the 550 to 5,000 pixel range analysis was typically low; ranging from 0 to 50 events, 51 to 100 events were observed in densely populated cores. Once the number of events obtained in the 550 to 5,000 pixel range analysis exceeded 100 events, the sample was excluded as the automated nuclei count became unreliable. To validate, the automated results generated were overlaid on the original image for 10 random images and an error margin <10% was determined (Table S2 in Additional file 3).

### Statistics

**Figure 1:**
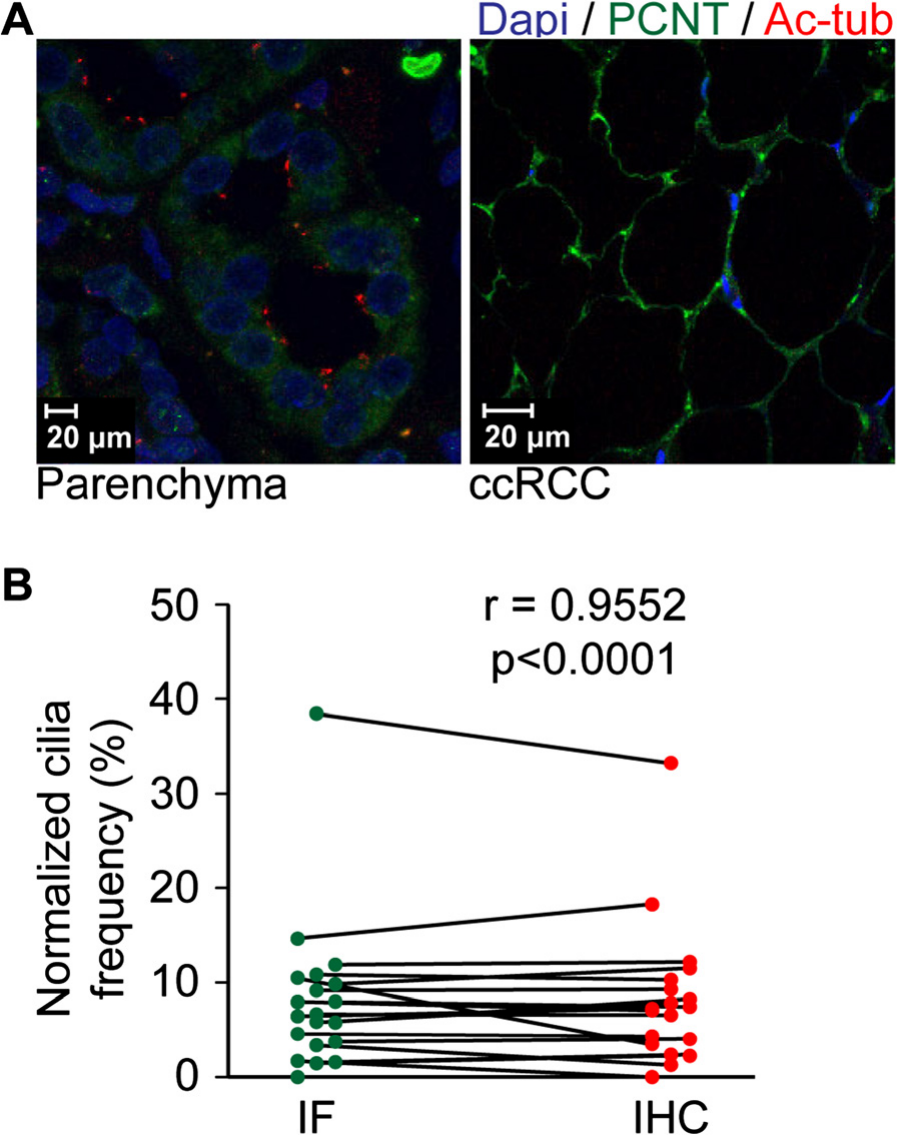
**Immunofluorescent analysis of cilia in renal tissues.** (**A**) Sections (4 μm) of renal parenchymal tissues and tumor tissues were stained with DAPI, acetylated-α-tubulin (Ac-tub) and pericentrin (PCNT) to mark cell nuclei and cilia. Presented images are maximal projections of confocal images of typical parenchymal tissue and a representative ccRCC. Scale bars 20 μm. (**B**) Normalized cilia frequencies of renal tumors, shown are paired quantifications of n = 20 samples. The plot compares the two cilia quantification methodologies described; data was obtained by immunofluorescent (IF) confocal image acquisition or scoring of immunohistochemical (IHC) stained sections. Statistics were determined by performing a paired *t*-test at a 95% confidence interval.

**Figure 2:**
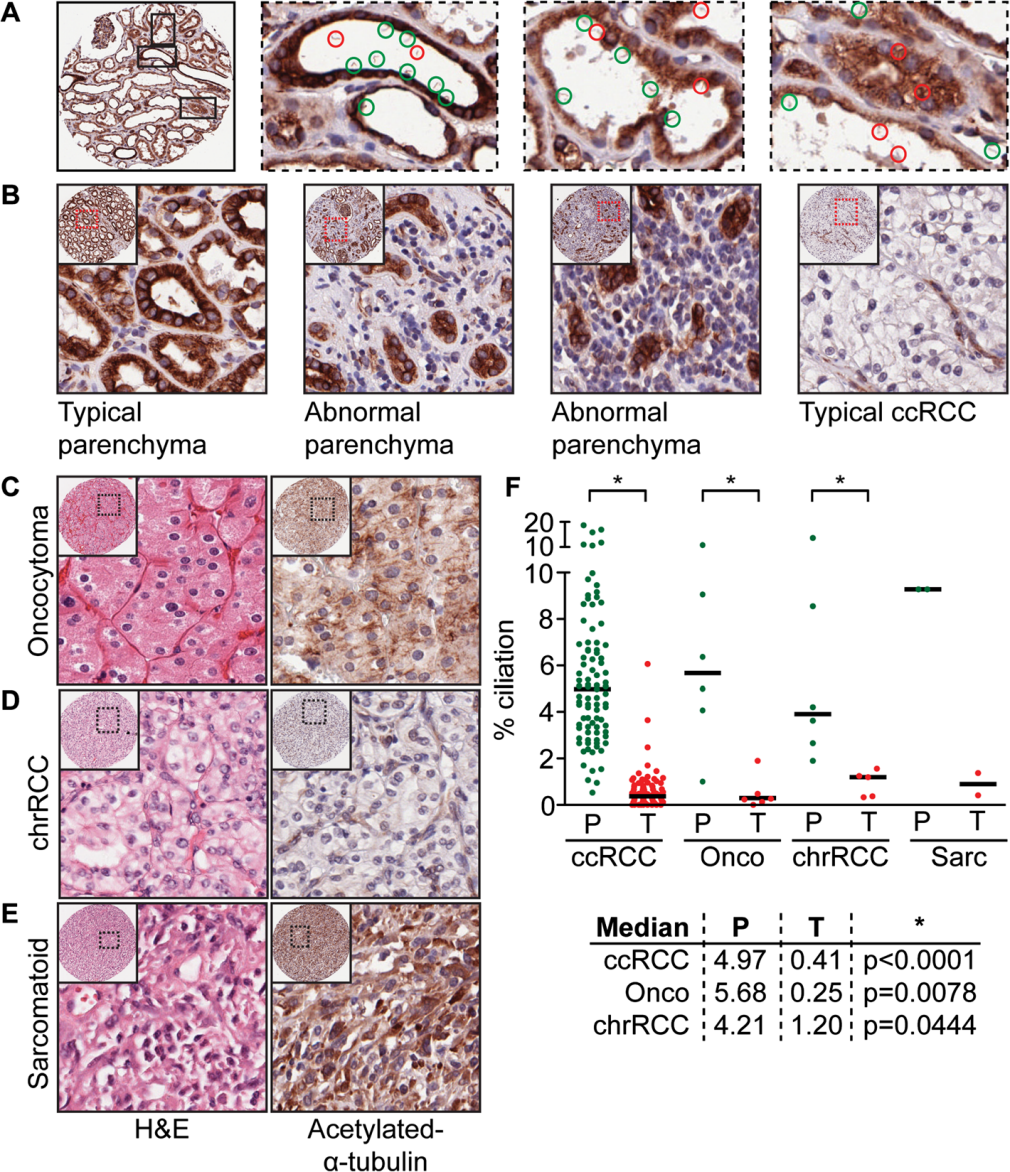
**Determination of cilia frequency.** (**A**) Acetylated-α-tubulin-stained renal tissue section obtained from the parenchyma of tumor tissue shows the presence of cilia lining tubular structures. Structures encircled in green are identified as cilia, red encircled structures are considered aberrant acetylated-α-tubulin staining or lack sufficient clarity. Stromal or supportive tissue cells between tubules typically do not have cilia. (**B**) Acetylated-α-tubulin-stained section of tissue obtained from typical and abnormal parenchymal tissue and a typical ccRCC. (**C-E**) Hematoxylin and eosin (H&E) and acetylated-α-tubulin stainings of a typical oncocytoma, chromophobe RCC (chrRCC) and sarcomatoid renal tumor. (**F**) Scatter plot and median of cilia frequency in parenchymal (P, green) tissues compared to matched RCC subtypes (T, red). Significant populations using *t*-test (95% confidence) are indicated with asterisks. All images 1 mm core and outlined magnifications.

For Figure 1B, we compared the normalized cilia frequencies of randomly chosen tumor tissues. Using a paired *t*-test analysis at 95% confidence (Prism5) we determined *P* <0.0001 and r = 0.9552 (n = 20). For Figure 2F, cilia frequencies were calculated as a percentage of cilia events compared to nuclei events. The averaged cilia frequencies of three core sections we compared between parenchymal tissue and tumor tissue. Using paired *t*-test analyses at 95% confidence (Prism5), the cilia frequencies of ccRCC, oncocytoma and chrRCC populations were determined at *P* <0.0001 (n = 89), *P* = 0.0078 (n = 6) and *P* = 0.0444 (n = 5) respectively.

## Results

### Classification of renal tumors

A collection of renal tumor biopsies and tissue of the tumor parenchyma, recovered 1 cm to 8 cm adjacent to the tumor lesion, was used to compile a TMA. Sections were previously well-documented and classified according to WHO standards by pathologists [23]. Both the tumor and parenchymal tissue were represented by three cores 1 mm in diameter. A total of eighty-nine clear cell RCC (ccRCC) of which ten were obtained from VHL patients, eight papillary RCC (pRCC), five chromophobe RCC (chrRCC), six oncocytomas and two sarcomatoid renal tumors and their corresponding parenchymal tissue were analyzed.

### Analysis of total cell numbers in normal and cancer tissue

In order to generate a percentage of ciliation per sample, the number of cells composing each core section was determined. For this, we developed a methodology to automatically count the number of nuclei per core (for an extended description see the Methods section). In brief, a colorimetric selection is set to match the nuclear marker hematoxylin and extracted to deplete background. Subsequently, images are further processed and analyzed using ImageJ particle analysis to determine the total nuclei number per core (Figure S1A, B in Additional file 2). Overlaying the identified nuclear events from the automated analysis with the original core for 10 samples of various pathologies indicates there to be an underestimation error that is <10% for ccRCC and chrRCC and oncocytoma (Figure S1C in Additional file 2, Table S2 in Additional file 3). pRCC cell nuclei are often intensely stained and aggregated, as the automated analysis exceeded our predetermined parameter threshold (see Methods) and rendered the automated quantification unreliable, we excluded these samples for automated nuclei count. It is important to emphasize that the acetylated-α-tubulin-stained TMAs that we use to count cilia frequencies were not suitable for automated nuclei count as the immunohistochemistry (IHC) interferes with the nuclear hematoxylin signal; we therefore used corresponding hematoxylin and eosin-stained TMA sections checked for matching morphology and cell counts.

### Cilia frequency in renal tumor cores

TMA sections were stained by immunofluorescence with acetylated-α-tubulin and pericentrin, established markers for the axoneme and base of the cilium, respectively. This approach allows sensitive high-resolution images of cilia to be captured and scored using confocal z-stack imaging (Figure 1A); however, for comparing a large number of patients systematically in a single experiment, we found that the imaging time required for each core (approximately 1 hour per patient) negatively affected subsequent sample signals through indirect bleaching, warming and aging. We therefore validated immunofluorescent high-resolution immunostaining in 20 random patients with standard immunochemistry for acetylated-α-tubulin. Standard IHC for acetylated-α-tubulin could obtain more data through semi-automatic processing; however, it was not clear that the lesser sensitivity to signal and 3D processing would produce ciliary frequency data comparable to immunofluorescence. Prior to manually counting cilia, parameters were established by which putative cilia were to be included or excluded: 1) size; the cilium needed to be a thin structure, intensely stained broad structures were considered background; 2) elongation; the cilium needed to be a continuous thin extending structure, square or dot-like structures were excluded. Because higher cilia counts overall were observed using the immunofluorescent stainings, we compared normalized data when the parenchymal tissue ciliation frequency was set to 100%. In this analysis, we observe an extremely strong correlation (r = 0.9552) between ciliary frequencies in all 20 samples comparing both techniques (Figure 1B).

We therefore felt confident that an approach involving IHC was justified and would yield relevant results in a greater number of patients that we could compare in a single experiment. Both staining protocols clearly mark cilia in luminal spaces in the normal tissues present on the TMA (Figures 1A and 2A). The TMA contains both tumor tissue and the parenchyma surrounding the tumor; three cores per tissue were scored. All cores were manually counted and the number of cilia per core was normalized to the number of nuclei as determined by the automated analysis, generating a percentage of ciliation per core (Table S1 in Additional file 1). The ciliation percentage was averaged for all three cores, we excluded cores that either generated nuclei numbers considered unreliable or showed visually aberrant acetylated-α-tubulin staining. In total, the TMA contained n = 89 sporadic or *VHL-*associated ccRCC (Figure 2B), n = 6 oncocytoma (Figure 2C), n = 5 chrRCC (Figure 2D) and n = 2 sarcomatoid renal tumor (Figure 2E) samples that were included in the cilia frequency analysis (Table S1 in Additional file 1). Sections of pRCC proved unsuitable for automated nuclei counting and on visual inspection of acetylated-α-tubulin-stained sections they appeared generally overstained and were not quantified. Of note, careful visual inspection of pRCC suggest this subtype to similarly exhibit reduced cilia numbers, although on occasion tubular structures can be identified that appear to contain cilia (Figure S2 in Additional file 4). We next compared the average ciliation frequencies of parenchymal tissue (P, in green) to their matched RCC subtype (T, in red) in a scatter plot (Figure 2E). Overall, ccRCC, oncocytoma, chrRCC and sarcomatoid renal tumors show a marked reduction in cilia frequency. A number of parenchymal samples appear to have cilia frequencies in the same range as tumor tissue samples, visual examination of these samples indicate that this parenchymal tissue was rather abnormal and contains either excessive stromal/supportive tissue or tumor cells (Figure 2B).

### Reduced ciliation in RCC subtypes is independent of cell proliferation

Cilia retraction occurs prior to cell duplication to allow for centrosome duplication and spindle pole formation, in early stages of the new cell cycle ciliogenesis is restored but restricted to cells that exit the cell cycle [26]. To ensure that the reduced cilia numbers are a characteristic of oncogenic transformation and exclude the possibility that this is due to an increase in proliferation, we performed antibody staining using the widely accepted proliferation marker Ki67. Three core tumor cores were blindly scored to determine the percentage of positively stained cells; results were averaged per sample. Except for the sarcomatoid tumor showing 23%, nearly all tumor samples had proliferation indexes, <5% (exceptions were a single VHL/ccRCC with 12% and a single chrRCC with 7.5%), which is too low to solely account for the observed loss of cilia (Table S1 in Additional file 1).

## Discussion

The effect of oncogenic transformation on cilia stability is tissue-specific and depends on the underlying mutation spectrum. It has been previously shown that cilia are less stable in early stages of melanoma and pancreatic ductal carcinoma development [27,28]. However, certain tumor types appear to depend on proper cilia formation, such as hedgehog-dependent basal cell carcinomas and medulloblastomas [29,30]. A number of tumor suppressor genes that have established roles in renal tumorigenesis, namely *VHL*, *TSC1*, *TSC2* and *FLCN*, also predispose to renal cyst formation, a common hallmark of ciliopathy syndromes. Indeed, these proteins have been shown to exert molecular activity towards cilia function. VHL interacts with the essential cilia microtubule motor complex kinesin-2 [31] and loss of cilia in mouse tissues is observed upon loss of *Vhlh* (the mouse ortholog of *VHL*) and either *Pten*[32] or *Gsk3β*[33]. We recently presented data concerning the role of FLCN in cilia formation show transiently reduced cilia formation in *FLCN-*depleted renal cells [34,35]. In contrast, loss of *Tsc1* or *Tsc2* enhances cilia length in mouse embryonic fibroblasts (MEFs) [36], and intriguingly, clinically there is a relatively low frequency of renal cyst and RCC formation in TSC patients [12]. Based on these observations we posed the question: to which extent is cilia frequency globally affected in RCC samples? We analyzed cilia frequency in renal tissue sections present in triplicate on a TMA of 110 patients, including RCC tissue and tissue obtained from the tumor parenchyma, and observed a severe reduction of cilia frequency in the various RCC subtypes. Our data supports, extends and confirms that the low ciliary frequency characteristic of renal cysts remains an evident characteristic of most renal tumors [22]. Potential effects on cilia function could not be analyzed in this approach, hence we cannot exclude whether cilia function in the normal tissue is also affected. Furthermore, the parenchymal tissue is likely also stressed and may very well be nonrepresentative of normal kidney function. Future analysis of downstream targets of signaling pathways common to cilia, such as the GLI3 and TCF/LEF transcription factors downstream of respectively the Shh and Wnt-pathways, might shed light on impaired cilia function in pretumorigenic tissue.

The underlying principle of cilia resorption exhibited by tumor cells and the putative necessity remains a highly debated topic. One possibility is that the loss of cilia promotes, or at least contributes to, an environment rendering these cells more susceptible to mitogenic cues that initiate proliferation. The mechanism is much more complex though, as classic ciliopathies (PKD, BBS, MKS, NPHP) do predispose to renal cysts, yet renal tumors are only rarely observed [20]. The best characterized functions of *VHL*, *TSC1*, *TSC2* and *FLCN* are also not directly related to cilia regulation, and RCC formation firstly depends on deregulated metabolic signaling [37]. The best characterized function for VHL is inactivation of HIF transcription factors in normoxic conditions through proteasomal degradation [38]. As a result of altered metabolic pathways, kidney cancers typically show a Warburg effect; where the use of glycolysis is promoted over mitochondrial oxidative phospholylation. All genetic factors contributing to hereditary kidney cancer (including MET, FH and SDH) described to date play regulatory roles in metabolic pathways regulated by nutrient-, oxygen-, iron- and energy-sensing. However, TSC1, TSC2 and FLCN have established roles in energy- and nutrient-sensing pathways through AMPK (5’-adenosine monophosphate-activated kinase) and downstream mTOR [37], and it is becoming evident that mTOR signaling and LKB1, an upstream kinase of AMPK, are both directly linked to cilia [39,40]. Combined, RCC seems to be a two-step process in which both the primary cilium function needs to be impaired as well as modification of the metabolic pathways and uncoupling regulation by energy, nutrients, oxygen and iron. Therapies aimed at restoring, stabilizing or molecularly circumventing cilia function might therefore be a suitable addition to current treatment strategies in an endeavor to prevent early events such as cyst formation and subsequent tumorigenic progression from this premalignant state [41].

## Conclusions

The crucial role of primary cilia in regulating renal homeostasis and the links to cell proliferation and polarization are intriguing facets in light of renal cancer research. In addition, findings that associate renal tumor suppressor proteins as ciliary functions regulators suggest that renal tumor cells preferentially are nonciliated. We therefore addressed the status of this organelle in RCC tissue. In this study, we included material from 110 patients of various RCC subtypes and compared three cores of RCC tissue to parenchymal renal tissue from the same patient. A severe reduction of cilia frequency in the various RCC subtypes is observed, which is independent of proliferation. Our results suggest that cilia loss is a common event in renal tumorigenesis and implies that cilia loss is part of a sequence of events leading to renal malignant tumor development.

## Abbreviations

ACKD: acquired cystic kidney disease; BBS: Bardet-Biedl syndrome; BHD: Birt-Hogg-Dubé syndrome; BSA: bovine serum albumin; ccRCC: clear cell RCC; chrRCC: chromophobe RCC; H&E: hematoxylin and eosin; IHC: immunohistochemistry; MEF: mouse embryonic fibroblast; MKS: Meckel-Gruber syndrome; NPHP: nephronophthisis; PBS: phosphate-buffered saline; PCNT: pericentrin; PKD: polycystic kidney disease; pRCC: papillary RCC; RCC: renal cell carcinoma; TMA: tissue microarray; TSC: tuberous sclerosis; UMCU: University Medical Center Utrecht; VHL: Von Hippel-Lindau disease.

## Competing interests

The authors declare that they have no competing interests.

## Authors’ contribution

SB and RG designed the research and SB, SW, GS and RG carried out the research. SB, SW, GS, JV and RG analyzed the data. SB, EV, PD and RG wrote the paper. All authors read and approved the final manuscript.

## Supplementary Material

Additional file 1 **Table S1.** Sample data of parenchymal and RCC tissues. Click here for file

Additional file 2 **Figure S1.** Automated nuclei count on HE-stained TMA. **(A)** Hematoxylin specifically marks cell nuclei, generating a dark-blue/purple color. In Photoshop, using the color picker, a color selection was matched to the hematoxylin signal (indicated in white boxes) and extracted. In ImageJ, the extracted image fragments are converted to 8-bit and made binary. **(B)** A total of three particle analyses are run per image, counting single (green), double (blue) and clustered cells (“>”, white) in the merged image, inverted separate analyses images shown for clarity. **(C)** Overlay of the particle analysis and the original image. Manual validation of accuracy was analyzed for 10 random samples (see Table S1). For clarity, the overlay with the original tissue is also shown in black and white; events not recognized by the automated nuclei count are indicated. Click here for file

Additional file 3 **Table S2.** Validation of automated nuclei count. Randomly selected sections (n = 10) of parenchymal and tumor tissues were analyzed by overlaying the original image with recovered particle analysis events. The number of nuclei that are not recognized by the automated methodology are used to calculate the percentage of error. The error typically indicates an underrepresentation. Click here for file

Additional file 4 **Figure S2.** Papillary RCCs are unsuitable for quantification. Three representative sections of papillary RCCs stained with hematoxylin and eosin (H&E) shows strongly stained and densely distributed nuclei. In accordance with our defined parameters, automated nuclei determination proved unreliable. Acetylated-α-tubulin staining shows intense levels in papillary RCC (pRCC) that renders them inadequate for reliable quantitative scoring. It can be appreciated that parts of the tissue has maintained some tubular structure and close observation incidentally shows cilia present (green circles), however our general impression is that cilia numbers are reduced in pRCC. Click here for file
