# Supplementary figures and images for: Reduced cilia frequencies in human renal cell carcinomas versus neighboring parenchymal tissue
Source: Cilia. 2013 Jan 31;2:2. doi: 10.1186/2046-2530-2-2 (PMC3564780; doi:10.1186/2046-2530-2-2)

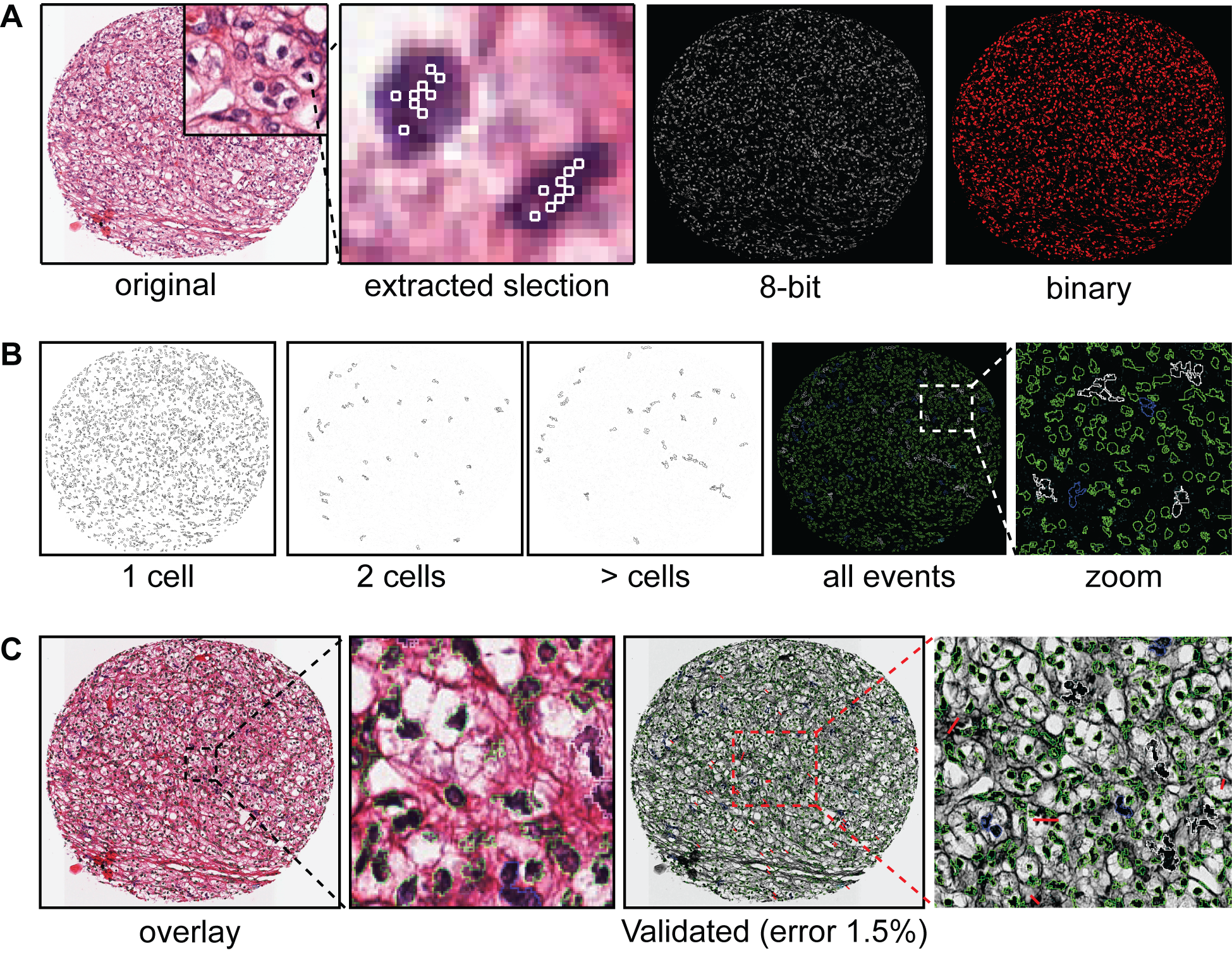

Supplement: Additional file 2 — Figure S1. Automated nuclei count on HE-stained TMA. (A) Hematoxylin specifically marks cell nuclei, generating a dark-blue/purple color. In Photoshop, using the color picker, a color selection was matched to the hematoxylin signal (indicated in white boxes) and extracted. In ImageJ, the extracted image fragments are converted to 8-bit and made binary. (B) A total of three particle analyses are run per image, counting single (green), double (blue) and clustered cells (“>”, white) in the merged image, inverted separate analyses images shown for clarity. (C) Overlay of the particle analysis and the original image. Manual validation of accuracy was analyzed for 10 random samples (see Table S1). For clarity, the overlay with the original tissue is also shown in black and white; events not recognized by the automated nuclei count are indicated. [file 2046-2530-2-2-S2.tiff]

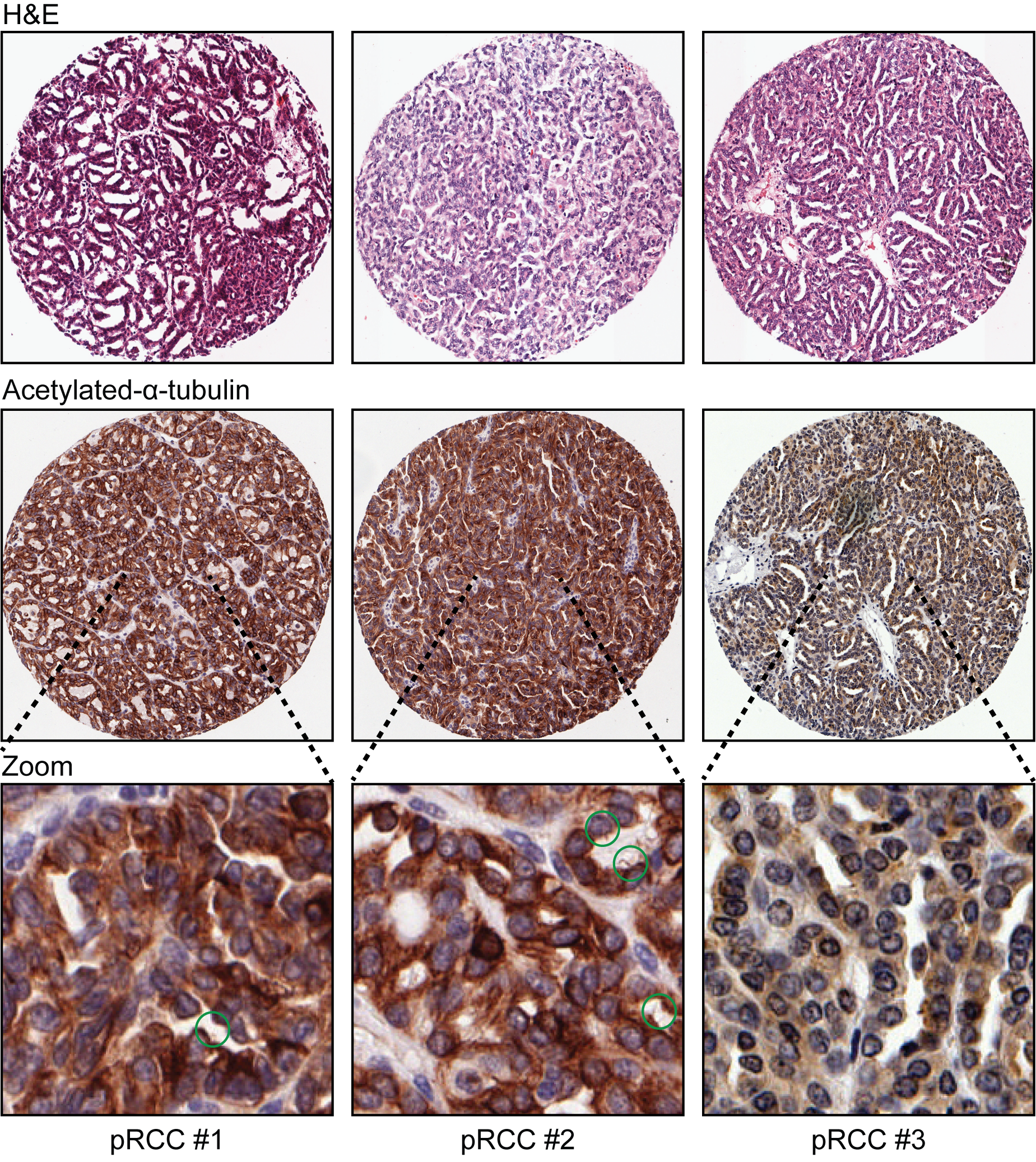

Supplement: Additional file 4 — Figure S2. Papillary RCCs are unsuitable for quantification. Three representative sections of papillary RCCs stained with hematoxylin and eosin (H&E) shows strongly stained and densely distributed nuclei. In accordance with our defined parameters, automated nuclei determination proved unreliable. Acetylated-α-tubulin staining shows intense levels in papillary RCC (pRCC) that renders them inadequate for reliable quantitative scoring. It can be appreciated that parts of the tissue has maintained some tubular structure and close observation incidentally shows cilia present (green circles), however our general impression is that cilia numbers are reduced in pRCC. [file 2046-2530-2-2-S4.tiff]
