# Supplementary material for: Reduced cilia frequencies in human renal cell carcinomas versus neighboring parenchymal tissue
Source: Cilia. 2013 Jan 31;2:2. doi: 10.1186/2046-2530-2-2 (PMC3564780; doi:10.1186/2046-2530-2-2)
Supplement: Additional file 3 — Table S2. Validation of automated nuclei count. Randomly selected sections (n = 10) of parenchymal and tumor tissues were analyzed by overlaying the original image with recovered particle analysis events. The number of nuclei that are not recognized by the automated methodology are used to calculate the percentage of error. The error typically indicates an underrepresentation. [file 2046-2530-2-2-S3.docx]

**Table S2: Validation of automated nuclei count**

Randomly selected sections (n=10) of parenchymal and tumor tissues were analyzed by overlaying the original image with recovered particle analysis events. The number of nuclei that are not recognized by the automated methodology are used to calculate the percentage of error. The error typically indicates an underrepresentation.

| **Total nuclei events (automated)** | **Validated error (manual)** | **RCC subtype** |
| --- | --- | --- |
| 1155 | 6.7% | Clear Cell |
| 749 | 4.7% | Parenchyma |
| 1988 | 2.2% | Clear Cell |
| 1349 | 2.3% | Parenchyma |
| Out of range | - | Papillary |
| 2020 | 1.5% | Clear Cell |
| 879 | 3.8% | Parenchyma |
| 1045 | 5.1% | Clear Cell |
| 885 | 9.3% | Parenchyma |
| 984 | 1% | Parenchyma |
